# Porcine xenotransplantation in the clinical era: converging advances and unresolved barriers on the path to clinical translation - a narrative review

**DOI:** 10.3389/fimmu.2026.1866007

**Published:** 2026-06-15

**Authors:** Douglas Henderson, Leonard Knoedler, Olivier Mathieu, Nathalie Rouas-Freiss, Curtis L. Cetrulo, Gilles Lemaitre, David M. Smadja, Nicolas O. Fortunel, Alexandre G. Lellouch

**Affiliations:** 1 Faculty of Medicine, Université Paris Cité, Paris, France; 2 Institut Hospitalo-Universitaire Reconnect, Lariboisière Hospital, Assistance Publique - Hopitaux de Paris, Paris, France; 3 Department of Oral and Maxillofacial Surgery, Charité – Universitätsmedizin Berlin, Corporate Member of Freie Universität Berlin and Humboldt-Universität zu Berlin, Berlin, Germany; 4 Department of Plastic and Reconstructive Surgery, Hôpital Saint-Louis, Assistance Publique – Hôpitaux de Paris, Paris, France; 5 Commissariat à l'Énergie Atomique et aux Énergies Alternatives (CEA), Direction de la Recherche Fondamentale (DRF)-Institut de Biologie François Jacob, Service de Recherches en Hémato-Immunologie, Hôpital Saint-Louis, Paris, France; 6 Institut National de la Santé et de la Recherche Médicale (INSERM) U1342, Institut de Recherche Saint-Louis, Université Paris Cité Paris, Paris, France; 7 Division of Plastic and Reconstructive Surgery, Cedars-Sinai Hospital, Los Angeles, CA, United States; 8 Vascularized Composite Allotransplantation Laboratory, Center for Transplantation Sciences, Massachusetts General Hospital, Harvard Medical School, Boston, MA, United States; 9 Université Evry/Paris-Saclay, Evry, France; 10 Commissariat à l'Énergie Atomique et aux Énergies Alternatives (CEA), Inserm, Institut de Biologie François Jacob, Département de Radiobiologie Cellulaire et Moléculaire, UMR Stabilité Génétique Cellules Souches et Radiations, Fontenay-aux-Roses, France; 11 Laboratoire de Régénération et Radiopathologies Cutanées (LR2C), Evry, France; 12 Université Paris Cité, Inserm, The Paris Cardiovascular Research Center, Team Endotheliopathy and Hemostasis Disorders, Paris, France; 13 AP-HP, Hôpital Européen Georges Pompidou, Hematology Department, Paris, France; 14 Université Paris-Saclay, Gif-sur-Yvette, France

**Keywords:** genetic engineering, immunosuppression, porcine allotransplantation, porcine xenotransplantation, rejection monitoring, translational research

## Abstract

The persistent shortage of donor organs has renewed interest in porcine xenotransplantation as a scalable alternative to human allotransplantation. Advances in genome engineering and immunomodulation have accelerated the field from experimental proof-of-concept toward early clinical translation. This review summarizes recent progress in donor pig genetic modification, targeted immunosuppressive strategies, surgical implementation, and rejection surveillance. Multigene-edited pigs lacking major carbohydrate xenoantigens and expressing human complement, coagulation, and cytoprotective regulators have substantially reduced hyperacute and acute vascular rejection. In parallel, costimulation blockade targeting the CD40/CD154 pathway has enabled prolonged graft survival in non-human primates and supported the first pig-to-human heart and kidney transplants. Improvements in organ preservation, recipient selection, and molecular monitoring, including circulating graft-derived DNA and multi-omic profiling, have further strengthened translational readiness. Complementary porcine models, including pig-to-pig transplantation and vascularized composite tissue transplantation, provide valuable platforms for studying tolerance induction, surgical refinement, and long-term graft biology. Human-to-pig chimerism has also been explored as a potential strategy to promote immune tolerance and improve graft compatibility. Despite these advances, major barriers remain, including delayed antibody-mediated rejection, coagulation dysregulation, innate immune activation, infectious safety, and regulatory challenges. Porcine xenotransplantation has now entered an early clinical era, with durable immune control and long-term safety representing the next decisive milestones.

## Graphical Abstract

**Figure:**
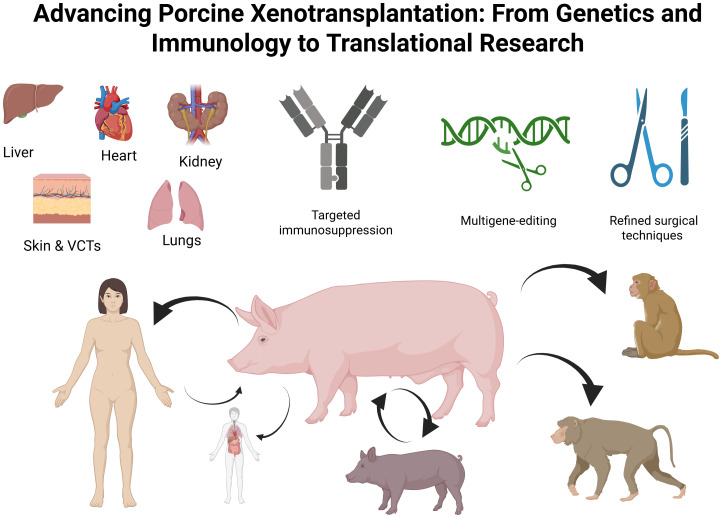
Advances in porcine xenotransplantation integrating multigene editing, targeted immunosuppression, refined surgery, and applications spanning solid organs and vascularized composite tissues (VCTs) toward clinical translation.

## 1 Introduction

The World Health Organization reports that, according to the most recent data from 2022 provided by the Global Observatory on Donation and Transplantation, over 150,000 solid organ transplants were performed globally, meeting no more than 10% of the estimated worldwide demand (1). As of September 2024, 89,792 patients in the United States were on the waiting list for kidney transplantation (2). In response to the growing shortage of human-donated solid organs available for transplantation, biomedical research has increasingly turned to animal models (3). Several mammalian species have been studied for xenotransplantation, including non-human primates (NHP), dogs, rabbits, rodents, and pigs (4). Among these, the porcine model offers numerous advantages.

Pigs exhibit anatomical and physiological similarities to humans, have rapid reproductive cycles, and are associated with fewer ethical constraints and lower costs than NHP (5, 6). Advances in genetic engineering and immunosuppression have markedly improved compatibility, reducing hyperacute and vascular rejection and extending graft survival in NHP models and initial human recipients (7). Surgical advances have enabled successful porcine heart, kidney, and liver xenotransplantation using optimized vascular and perfusion techniques (8–10).

Porcine xenotransplantation remains at a pivotal translational juncture, where experimental success must align with immunological safety and ethical acceptability (11–13). The porcine model integrates advances in genome editing, immunosuppression, and surgical innovation (14–17). As suggested in the case of heart failure, genetically engineered pig xenotransplantation may be combined with stem cell therapies and bioprosthetic artificial organs within integrated strategies (18). Furthermore, the porcine model significantly contributes to addressing the major challenges of immunological tolerance, ethical concerns, and cost-effectiveness currently faced in the field of vascularized composite allotransplantation (VCA). In light of these developments, a critical synthesis of recent data is warranted to define remaining barriers and guide future clinical translation. This review prioritizes landmark translational studies and clinical applicability, with mechanistic immunology being addressed where directly relevant to clinical outcomes.

**Figure 1:**
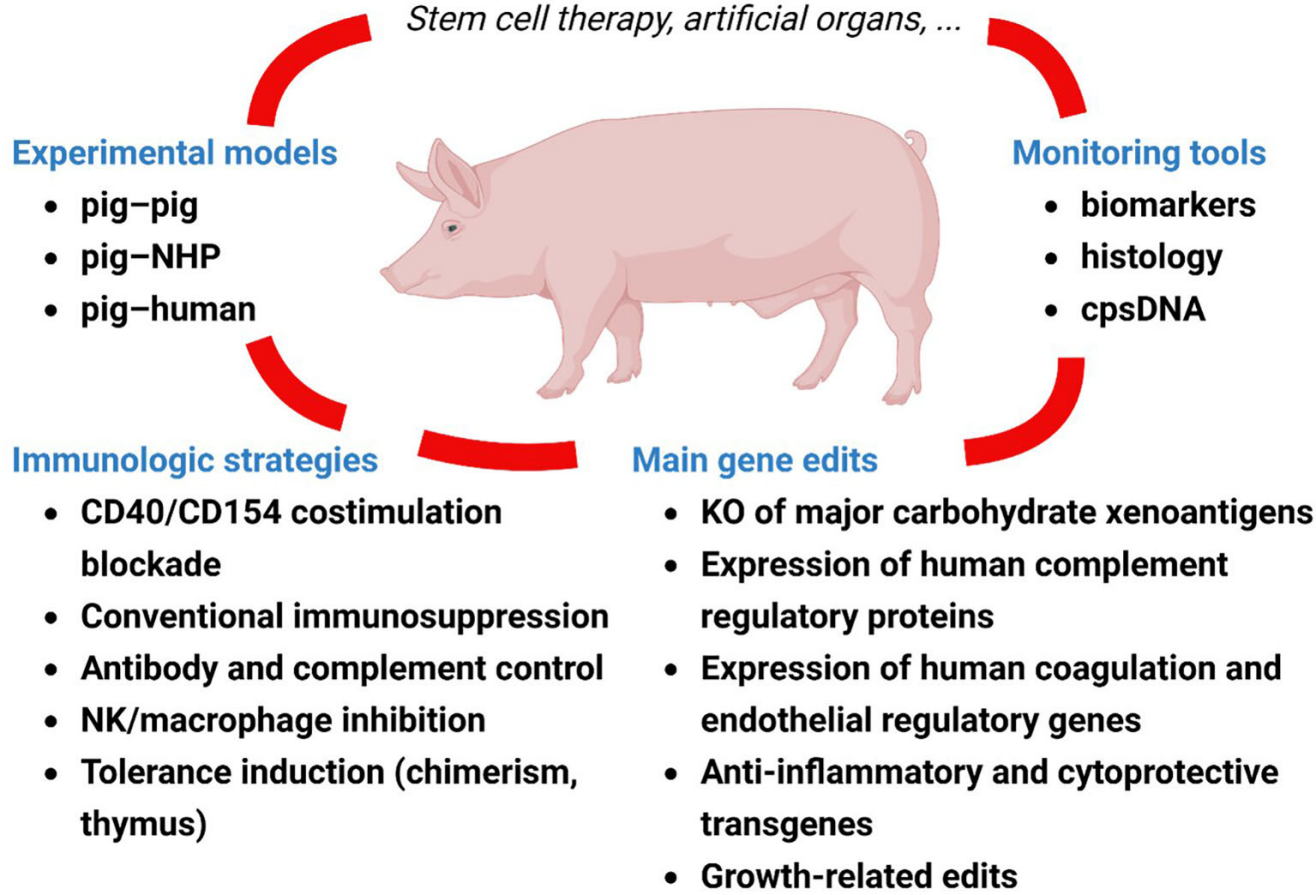
Translational framework of porcine xenotransplantation. Schematic overview of the various steps needed to overcome the porcine xenotransplantation challenges.

The aim of this review is to synthesize recent advances in porcine transplantation science, with xenotransplantation as the primary focus, while examining pig-to-pig allotransplantation and human-to-pig chimerism as complementary experimental models that may help clarify remaining immunological and physiological challenges. The translational framework structuring this review is summarized in **Figure 1**.

## 2 Methods

This narrative review of the literature was conducted by searching PubMed, Scopus, and Web of Science databases until May 2026. Keywords, along with their synonyms and corresponding MeSH terms, were combined using Boolean operators (AND/OR); e.g., pig, xenograft, allograft, chimerism, pig-to-pig, pig-to-human, human-to-pig, rejection, vascularized composite tissue. Only articles published in English that were published in peer-reviewed journals were considered eligible. Case reports, original research articles, systematic reviews, and expert consensus statements were included when directly relevant to the scope of this review. Given the narrative design, no formal PROSPERO registration was undertaken; however, inclusion and exclusion criteria were predefined by the study team prior to screening. As this study is not a systematic review, no formal study count or PRISMA flow diagram is provided. Among the numerous search results identified, articles were selected based on clinical relevance, translational importance, methodological robustness, and recency. The selected papers were subsequently categorized thematically by two reviewers into the following domains: genetic engineering, immunosuppression, surgical techniques, rejection monitoring, and translational and ethical considerations.

## 3 Porcine models in transplantation research

To structure this review, we underline three persistent translational challenges that emerge across models (1): incomplete control of innate and residual adaptive immune pathways (2); physiological incompatibilities affecting renal, cardiac, and hepatic function; and (3) gaps between preclinical modeling and early human experience.

### 3.1 General considerations

Pigs and humans share important molecular, cellular, and anatomical similarities, making swine a valuable biomedical model (5). A major advantage is the high sequence and chromosomal structural homology between pig and human genomes (19). Across 17 homologous tissue types, 15,944 one-to-one orthologous genes accounted for approximately 82% and 87% of total gene expression in pigs and humans, respectively. Compared with rats, mice, cats, or cattle, pigs show greater genetic and regulatory similarity to humans, although this remains lower than in NHP, such as the chimpanzee (20, 21). Based on analyzed immunological parameters, the porcine immune system resembles the human immune system by over 80%, compared with about 10% for mice (22). Physiologically, pigs may also be suitable for pharmacological studies of renal drug elimination (23). In cardiovascular research, the pig heart-to-body weight ratio (~5 g/kg) closely matches that of adult humans, compared with ~7 g/kg in dogs and ~3 g/kg in sheep (24). Pigs are also increasingly used in neuroscience because of their anatomical and functional brain characteristics (25). Moreover, single-cell and spatial transcriptomic studies of fetal pig skin showed close resemblance to human epidermal architecture and gene expression (26).

Historically, xenogeneic tissues have been used clinically for decades in bioprosthetic heart valves (BHV) (27). These valves are derived from porcine or bovine tissues and rendered nonviable by glutaraldehyde fixation, but they may not be immunologically inert (28). Residual carbohydrate xenoantigens have been implicated in structural valve degeneration. Neu5Gc persists in tested commercial BHV and is recognized by human anti-Neu5Gc IgG (29). In a rat implantation model, α-Gal–positive BHV showed greater calcification than α-Gal–deficient tissues, and preformed anti-Gal antibodies accelerated this process (30). Host factors may also contribute: blood group A has been associated with more pannus formation and leaflet tears, whereas blood group B has been linked to increased calcification and perforation (31). These findings may provide valuable historical and mechanistic insights for contemporary porcine xenotransplantation strategies.

From an ethical perspective, the use of pigs raises fewer concerns than that of NHP. Given their domesticated status, pigs are more amenable to controlled breeding and husbandry conditions. Moreover, the close genetic proximity of NHP to humans might increase the likelihood of cross-species disease transmission (32). Accordingly, some countries, including the United Kingdom, have restricted the use of NHP as transplant sources (6). Pigs may also face less societal opposition because they are widely raised for food in many regions (33). In addition, domestic pigs reach sexual maturity at six to eight months, reproduce year-round, and have a short gestation period of about four months (34). These rapid reproductive characteristics significantly influence the financial cost of research and may enable a timely response to the needs of patients suffering from severe organ failure. Combined with their biological similarities to humans, these features make pigs a preferred model for xenotransplantation.

### 3.2 Development and refinement of surgical techniques

Xenotransplantation of porcine organs into humans has required major anatomical and surgical refinement. Detailed knowledge of porcine cardiac anatomy is essential for donor retrieval and implantation, together with thorough preoperative imaging (9). The feasibility of porcine aortic valve transplantation has been demonstrated in a cadaveric model, offering a potential alternative to conventional prostheses that lack growth potential in neonates and infants, particularly when anticoagulation is contraindicated (35). In liver xenotransplantation, heterotopic implantation of a porcine liver without removing the native liver has been proposed as a temporary bridge for acute liver failure while awaiting allotransplantation or recovery (8). In addition, porcine kidney xenotransplantation in two brain-dead human recipients remained viable for at least 54 hours using standard vascular anastomoses (10). These early results have led some experts to support pilot clinical trials in living recipients (36). Overall, continued progress requires close collaboration between surgeons and translational researchers.

### 3.3 Pig-to-pig allotransplantation research models

Pig-to-pig allotransplantation is an important intermediate model toward successful pig-to-human xenotransplantation. A reproducible kidney transplant model in low-weight miniature pigs has been developed for preclinical studies and refinement of vascular and ureteral anastomoses while maintaining reliable graft function (37). A porcine lung allotransplant model reproduces early human post-transplant physiology and is useful for studying primary graft dysfunction and ischemia–reperfusion injury (38). Normothermic ex vivo perfusion has also been used to preserve donor pig hearts before heterotopic intra-abdominal transplantation, enabling prolonged graft viability (39). Tolerance studies in miniature swine have shown donor-specific acceptance of cardiac, skeletal, and musculoskeletal allografts, although skin components remain more immunogenic and prone to rejection (40–42). In VCA, heterotopic hemifacial and auricular transplant models have enabled serial biopsies, immune monitoring, and assessment of compatibility strategies, with encouraging short-term graft survival (43, 44). These advances establish pig allotransplantation as a robust translational platform. Furthermore, it uniquely enables the analysis of surgical variables, tolerance-induction strategies, and early physiological incompatibilities within a controlled genetic background, factors that are difficult to isolate in pig-to-NHP or pig-to-human systems.

### 3.4 Pig-to–NHP xenotransplantation research models

Before initiating human clinical trials, immunosuppressive protocols and surgical techniques should be evaluated in pig-to-primate or NHP xenotransplantation models. In a pig-to-rhesus macaque kidney model, long-term survival beyond one year was achieved by selecting recipients with low anti-pig antibodies and depleting CD4^+^ T cells (45). Genetically engineered pig hearts with ten gene edits were transplanted into baboons and functioned for up to 225 days, showing resistance to early rejection (46). The pig-to-NHP model remains critical. Cooper et al. proposed that, before clinical trials, reproducible recipient survival of at least six months after vital organ transplantation (heart or kidney) should be demonstrated in five to ten consecutive experiments (47). However, the predictive value of NHP models is imperfect, as primate antibody repertoires, innate immunity, and coagulation differ from humans, so success in NHPs does not always predict human outcomes. A carefully staged transition from animal studies to clinical use is therefore essential. NHP models also do not fully reproduce human xenoreactivity, particularly regarding carbohydrate antigen expression. While early long-term graft survival was achieved in NHPs, these models did not require the same level of xenoantigen elimination needed in humans. In contrast, successful clinical translation required triple-knockout (KO) donor pigs (GGTA1, CMAH, and B4GALNT2), highlighting key interspecies differences, especially with Old World primates (48, 49). This discrepancy contributed to the delay between preclinical success and initiation of human clinical trials. Importantly, the genetic background of donor pigs used in these successful NHP studies closely informed subsequent clinical applications, with similar multigene-edited donor lines later employed in human kidney xenotransplantation.

### 3.5 Experimental models of pig-to-human xenotransplantation

Since 2022, porcine heart, kidney, liver, and lung xenotransplantation into humans has been reported. Early clinical experience may be less a validation of NHP models than an opportunity to determine which preclinical findings are truly translatable. The first cardiac xenotransplant was performed on a compassionate-use basis in a patient supported by extracorporeal membrane oxygenation. The graft came from a ten-gene-edited pig with KO of GGTA1, CMAH, B4GALNT2, and GHR, plus six human transgenes (CD46, CD55, THBD, PROCR, CD47, HMOX1) to improve immune, inflammatory, and coagulation compatibility. The graft maintained circulatory function for seven weeks (50). Genetically modified pig kidneys were also transplanted into two brain-dead human recipients. During 54 hours of follow-up, urine output and filtration improved, and serial biopsies showed no acute rejection (10). A minimally gene-edited pig thymokidney (GGTA1 KO) sustained life-supporting human renal function for 61 days in a decedent model (51). A six-gene-edited pig liver was transplanted into a brain-dead recipient and remained functional for 10 days, producing bile and proteins without hyperacute rejection. Hemodynamic and coagulation parameters remained stable, with no evidence of porcine viral transmission (52). A 10-gene-edited pig liver was transplanted heterotopically in a 71-year-old patient with hepatitis B-related cirrhosis and unresectable hepatocellular carcinoma. No hyperacute or acute rejection was observed during the first 31 days, porcine albumin and coagulation factors were detected in the recipient’s blood, reflecting metabolic and synthetic function of the graft. Thrombotic microangiopathy (TMA) led to graft removal on postoperative day 38 and was subsequently controlled by eculizumab and plasma exchange; the patient survived 171 days (53). Overall, genetically modified porcine hearts, kidneys, and livers have shown promising short-term graft function without hyperacute rejection. Meaningful success should be judged not only by survival duration, but also by physiological integration, absence of consumptive coagulopathy, and sustained immune control under clinically manageable immunosuppression. Although brain-dead recipient models have provided valuable physiological and immunological insights, their relevance remains debated because of altered systemic conditions and the lack of long-term host–graft interactions. Extrapolation to living recipients should therefore be cautious (54). **Table 1** provides an overview of selected reported porcine solid-organ xenotransplants performed in humans, including procedures in both living recipients and brain-dead decedents. It should be noted that several additional cases, predominantly renal xenotransplants, have been reported in the public domain but have not yet been published in peer-reviewed journals (55).

**Table 1:** Overview of reported porcine-to-human xenotransplantation cases (2022–2026).

Organ	Year	Model	Genomic edits	Immunosuppression	Graft survival	Outcome/limitation
Kidney (10)	2022	Brain-dead decedent	GE=1: GGTA1 KO + vascularized thymic tissue (“thymokidney”)	Steroids + mycophenolate	54 hours	Immediate urine production; improved creatinine/eGFR; no hyperacute rejection
Heart (50)	2022	**Living patient**	GE=10: 3 KO (GGTA1, CMAH, B4GALNT2) + GHR KO + 6 human transgenes (CD46, CD55, THBD/TBM, PROCR/EPCR, CD47, HMOX1/HO-1)	CD40 blockade (KPL-404) + rituximab + antithymocyte globulin + steroids + mycophenolate (later tacrolimus) + complement inhibition	49 days primary failure/60 days total support	Successfully weaned from ECMO; normal early graft function without apparent rejection. Sudden diastolic thickening/failure on day 49; life support withdrawn day 60.
Heart (83)	2023	Brain-dead decedents	GE=10: 4 KO (GGTA1, CMAH, B4GALNT2, GHR) + 6 human transgenes (THBD/TBM, CD46, CD55/DAF, PROCR/EPCR, HMOX1/HO-1, CD59)	Induction: rabbit ATG, methylprednisolone, eculizumab. Maintenance: high-dose steroids + mycophenolate mofetil; heparin infusions	66 hours (study endpoint)	Both grafts functioned immediately with no hyperacute, cellular, or antibody-mediated rejection. Recipient 1 developed progressive dysfunction likely due to donor–recipient size mismatch and ischemic injury; Recipient 2 remained stable with preserved function. decedent model, no long-term immune assessment
Kidney (74)	2024	**Living patient**	Genomic sites Edited=69: 3KO (GGTA1/CMAH/B4GALNT2) + PERV A/B/C inactivation + 7 human transgenes (TNFAIP3, HMOX1/HO-1, CD47, CD46, CD55/DAF, THBD/TBM, PROCR/EPCR)	ATG + rituximab + tegoprubart + ravulizumab; tacrolimus + mycophenolic acid + prednisone	52 days (censored at patient death)	Immediate urine production; dialysis stopped; creatinine 11.8→2.2 mg/dL by day 6; day-8 acute T-cell rejection reversed; recipient died suddenly of cardiac causes on day 52
Liver (52)	2025	Brain-dead decedent	GE=6: 3KO (GGTA1, B4GALNT2, CMAH) + human transgenes (CD46, CD55/DAF, THBD/TBM)	Pre-op: ATG, eculizumab. Post-op: methylprednisolone, tacrolimus, MMF, intermittent etanercept; rituximab introduced from day 3 when B-cell activation increased	10 days	Graft remained functional until study completion; bile production began 2 h after reperfusion and reached 66.5 mL by POD10; porcine albumin increased; no histologic rejection.
Liver (53)	2025	**Living patient**	GE=10: 3 KO (GGTA1, CMAH, B4GALNT2) + 7 human transgenes (CD46, CD55/DAF, CD59, CD39, THBD/TBM, PROCR/EPCR, CD47)	Induction: rituximab, basiliximab, methylprednisolone Post-op tacrolimus, sirolimus, MMF, steroids; additional basiliximab and rituximab doses; eculizumab for TMA	38 days (graft removed due to TMA); patient survived 171 days	First pig-to-living-patient liver xenotransplant (auxiliary/heterotopic). No hyperacute or acute rejection in first 31 days; porcine albumin and coagulation factors functional. TMA on POD38 led to graft removal; managed with eculizumab and plasma exchange.
Kidney (51)	2025	Decedent (perfused model)	GE=1: GGTA1 KO + vascularized thymic tissue (“thymokidney”)	Induction: rATG, rituximab, methylprednisolone. Maintenance: prednisone, mycophenolate mofetil, tacrolimus, belatacept, eculizumab. Rejection treatment: plasma exchange, pegcetacoplan (C3/C3b inhibitor), pulse steroids, additional rATG	61 days (planned termination)	Immediate urine production; dialysis independence; stable hemodynamics/electrolytes. Excellent renal function until POD33, when antibody-mediated rejection occurred with rise in creatinine and donor-specific IgG. Rejection fully reversed with therapy; renal function returned to baseline.
Heart (70)	2025	**Living patient**	GE=10: 4 KO (GGTA1, CMAH, B4GALNT2, GHR) + 6 human transgenes (THBD/TBM, PROCR/EPCR, CD46, DAF/CD55, HMOX1/HO-1, CD47)	Costimulation blockade centered regimen. Anti-CD40L monoclonal antibody Tegoprubart (induction + maintenance), rATG lymphodepletion, rituximab, corticosteroids, complement inhibition first with C1 esterase inhibitor, eculizumab (C5 blockade), later carfilzomib + therapeutic plasma exchange	40 days (comfort care on POD40; ECMO from POD31)	Initial graft function excellent with EF 55-65%, no inotropic support early, marked improvement in heart failure symptoms. By POD13 biopsy showed endothelial activation with IgG/IgM/C3d/C4d deposition suggesting early AMR. Rapid progression around POD29–31 to restrictive/diastolic failure, wall thickening, then severe systolic dysfunction
Kidney (122, 123, 129, 130)	2025	**Living patient** (UKidney trial, FDA)	Genomic sites Edited=69: 3 xenoantigen KO (GTKO/GGTA1, B4GALNT2, CMAH), PERV A/B/C inactivation, 7 human transgenes (CD46, CD55/DAF, PROCR/EPCR, THBD/TBM, CD47, HMOX1/HO1, A20)	Induction: ATG, anti-CD20 antibody, C3 inhibitor. Maintenance: Fc-silent anti-CD154 agent (costimulation blockade), tacrolimus, MMF, steroids	271 days (record survival; graft later explanted due to persistent proteinuria)	Immediate graft function without prolonged dialysis; patient regained energy, improved quality of life, normal metabolic filtration during functional period, reduced dialysis dependence. Landmark longest pig-to-human solid organ survival.
Lung (131)	2025	Brain-dead recipient	GE=6: 3KO (GGTA1, B4GALNT2, CMAH) + 3 human transgenes (CD46, CD55/DAF, THBD/TBM)	rATG, basiliximab, rituximab, eculizumab, tofacitinib, tacrolimus, MMF, steroids, belatacept	216 h (9 days)	No hyperacute rejection; viable/functioning graft. PGD-like edema at 24 h; AMR on POD3/6 with partial recovery POD9; no uncontrolled infection

Bold values indicate xenotransplantation cases performed in living recipients.

### 3.6 Human-to-pig chimerism and ethical challenges

Another way to enhance xenograft tolerance could be through the creation of human-to-pig chimeras. In a human-to-pig spleen xenotransplantation model, human spleen slices were transplanted into cyclophosphamide-immunosuppressed pigs. Transient human cell chimerism was detected in porcine peripheral blood, showing two peaks at approximately day 2 and day 14 post-transplantation. These fluctuations paralleled changes in cytotoxicity, with both graft-versus-host and host-versus-graft responses (56). Using a minimally invasive, ultrasound-guided *in utero* injection of human umbilical cord blood mononuclear or bone marrow–derived mesenchymal stem cells, other researchers generated human–pig chimeric piglets. Human cell chimerism was confirmed in up to 21% of piglets by flow cytometry and 51% by quantitative PCR, with evidence of inter-fetal and maternal cell trafficking (57). It should be noted that chimerism, in the context of transplantation tolerance, refers specifically to the sustained presence of donor-derived cells within the host, rather than the expression of donor-derived proteins alone. While human–pig chimerism may enhance tolerance of porcine xenografts, it raises novel ethical questions (e.g., Does introducing human cells into pigs change the animal’s moral or legal status)?. Several authors suggest that such research should proceed under rigorous oversight, transparent governance, and safeguards against any unacceptable humanization of the animal host (58–60).

### 3.7 Fundamental immunosuppression strategies in pigs

Innate immune activation represents a persistent barrier. Macrophage polarization toward a pro-inflammatory phenotype upon contact with porcine endothelium contributes to early graft injury (61). NETosis has been implicated in endothelial injury and neutrophil-mediated xenogeneic cytotoxicity in xenotransplantation models (62). Innate immune amplification involves endothelial activation, complement/coagulation cascades, and inflammatory innate immune signaling pathways (63).

Over the past four decades, pig to NHP organ xenotransplantation has advanced markedly through genetic engineering and immunosuppression. Early attempts were limited by hyperacute rejection driven by natural antibodies and complement activation, but pigs lacking the three major glycan xenoantigens and expressing human complement, coagulation, and anti-inflammatory regulators have greatly improved outcomes (64). Immunosuppressive protocols targeting the CD40/CD154 co stimulation pathway, combined with anti-inflammatory adjuncts, have effectively prevented rejection with acceptable safety profiles (65). Conventional regimens include tacrolimus, mycophenolate mofetil, and corticosteroids (66). Additional therapies such as anti-complement agents and plasmapheresis help control humoral rejection (67). Emerging tolerance strategies, including thymic transplantation and mixed chimerism, remain promising (68).

In a pivotal study, six consecutive porcine to NHP kidney xenotransplants using pigs with ten genetic edits achieved a mean survival of 220 days (median 261 days). Most recipients received anti-CD40–based costimulatory blockade, and low-risk primates maintained stable graft function beyond the early postoperative period (69). A recent opinion paper assessing clinical readiness emphasized rejection prevention. While heart xenotransplants achieved 61 and 40-day survival under emergency eINDs (electronic Investigational New Drug application, FDA authorization for human clinical trials) (50, 70), the authors argue renal xenografts hold greater promise for routine application (71).

Nevertheless, challenges remain: graft loss in approximately 40% of primates within three months (14), sensitization, physiological mismatch, infection safety, and regulatory barriers. Although progress supports early-phase human trials in end-stage renal disease, careful patient selection and immunological monitoring are essential (36, 71–73). Persistent issues include macrophage-mediated inflammation, innate immune activation, coagulation dysregulation, delayed xenograft dysfunction, and the need for complement inhibition, particularly of the C5 pathway (74), to reduce early antibody-mediated injury and improve graft survival.

### 3.8 Genetic engineering strategies in donor pigs

Genetic engineering of pigs is a central strategy to reduce immunogenicity, study immune barriers *in vivo*, and improve graft tolerance in xenotransplantation (75). Genome-editing tools such as CRISPR/Cas9, zinc finger nucleases, and TALENs allow precise modification of porcine genomes. Major approaches include KO of xenoantigens such as GGTA1, CMAH, and B4GALNT2 to prevent hyperacute and acute vascular rejection (76, 77), together with insertion of human transgenes (e.g., CD46, CD55, CD59, THBD) to regulate complement activation, coagulation, and cellular immune responses (78, 79). Inactivation of porcine endogenous retroviruses (PERVs) may also reduce zoonotic risk. Preclinical NHP studies have demonstrated prolonged graft survival and improved function (7). CRISPR-mediated inactivation of PERVs in pigs, as implemented in the 69-genomic-edit eGenesis pig (74), was developed to reduce the risk of pig-to-human viral transmission in xenotransplantation (80). However, challenges remain in establishing reliable post-transplant surveillance strategies for PERVs and other porcine pathogens such as porcine cytomegalovirus and hepatitis E virus (81).

A 2024 review proposed a “minimum essential” engineering strategy focused on preventing hyperacute rejection and coagulation dysfunction. Core modifications include deletion of carbohydrate xenoantigens and expression of human complement regulators, thromboregulatory factors (e.g., THBD, PROCR), with optional addition of CD47, TNFAIP3, and HMOX1 to enhance graft resilience (82). Notably, ten-gene-modified hearts and kidneys have recently been transplanted into brain-dead human recipients, with graft function reported for up to 66 hours for hearts and 7 days for kidneys (83, 84). Further advances include deep phenotyping of xenografts in perfused human cadavers to guide gene-edit selection, and transient SLA knockdown during ex vivo perfusion to further reduce immune recognition (85, 86).

These developments mark a significant step toward clinical application but also raise strategic questions regarding the balance between “minimum essential” edits and increasingly complex multigene configurations. While additional edits may incrementally reduce immunological barriers, they may also introduce functional uncertainty and diminishing translational returns, particularly in long-term human applications.

## 4 Objective assessment of rejection and immune tolerance states in porcine xenografts

### 4.1 Hematological and biological parameters

To monitor the tolerance of a porcine xenograft and the recipient’s immune acceptance, it is key to identify specific biological and histological markers. Certain markers are generic, whereas others exhibit species or organ specificity. In baboon models of pig-to-NHP renal and cardiac xenotransplantation, key biomarkers predictive of impending graft failure include serial declines in platelet counts and plasma fibrinogen levels, alongside elevations in inflammatory markers such as C-reactive protein and cytokines. These parameters, together with dynamic measures of systemic inflammation, provide early and robust indicators of rejection (87). In pig-to-NHP kidney xenotransplantation, serum creatinine and measured glomerular filtration rate reveal global graft performance, while new-onset proteinuria reflects early glomerular injury (88, 89). Serum electrolytes (sodium, potassium, chloride), acid–base status, calcium–phosphate balance, and plasma renin activity monitor homeostasis and uncover physiological mismatches, particularly in the renin–angiotensin–aldosterone and antidiuretic hormone systems. Anemia, often seen post-transplant, may reflect inadequate porcine erythropoietin stimulation of the recipient’s erythropoiesis (90). Hypercalcemia and hypophosphatemia, have also been reported in NHP xenotransplantation models, reflecting species-specific metabolic incompatibilities (91). In a pig-to-NHP corneal xenotransplantation study, early immune activation markers proved predictive of graft rejection: notably, an elevated proportion of circulating CD8^+^IFN-γ^+^ T-cells at week 2 and increased complement C3a levels in aqueous humor at week 4 (92). A multi-omic approach revealed the sequential activation of innate and adaptive human immune responses driving antibody- and T-cell–mediated rejection in a pig-to-human kidney xenotransplant, while identifying several early molecular predictors, such as CXCL9/10/11 signaling, complement activation, and plasmablast expansion (93).

### 4.2 Lymphocyte activation parameters

Experimental xenotransplantation using porcine organs has advanced significantly with genetic modifications mitigating hyperacute and acute vascular rejection; however, delayed xenograft rejection driven by cytotoxic lymphocytes remains a major barrier. Activation of human NK cells and CD8+ cytotoxic T lymphocytes against porcine endothelial cells involves complex receptor-ligand interactions, adhesion molecules, and both direct and indirect antigen recognition pathways (94, 95).

Strategies such as expression of human non classical MHC molecules (HLA-E, HLA-G), inhibitory ligands (PD-L1, encoded by CD274), and costimulatory blockade (CTLA4-Ig, LEA29Y) have been explored to inhibit lymphocyte activation (96–98). Costimulatory blockade, particularly targeting the CD40/CD154 pathway, remains one of the most effective strategies. Novel approaches, including multi-omics analyses (99) and thymic transplantation (100), aim to promote tolerance while minimizing systemic immunosuppression. Despite promising *in vitro* and ex vivo results, further research is essential to achieve comprehensive control of human lymphocyte responses.

### 4.3 Targeted immunosuppressive protocols

Over almost four decades of pig-to-NHP xenotransplant research, immunosuppressive strategies have evolved from conventional regimens, including cyclosporine, steroids, azathioprine or MMF, splenectomy, plasmapheresis and anti-Gal immunoadsorption (selective removal of natural anti-Gal antibodies from recipient blood), to sophisticated protocols that integrate gene-edited donor pigs with targeted immune modulation (65).

Pre-transplant antibody removal is complemented by peri-operative B-cell depletion (e.g., rituximab) (101), T-cell depletion (anti-thymocyte globulin), and, critically, co-stimulation blockade via anti-CD40/CD154 monoclonal antibodies (102). This approach reduces hyperacute and acute rejection while minimizing thrombosis risks associated with earlier anti-CD154 agents. Adjunctive therapies (i.e., anti-inflammatory agents, complement inhibitors, and anticoagulation) address systemic inflammation and proteinuria (65). Careful modulation of these strategies is critical, as concurrent use of certain agents (e.g., calcineurin inhibitors with co-stimulation blockers) may be harmful due to potential toxicity and complex immunological interaction.

### 4.4 Graft histology for the assessment of acute rejection and chronic pathology

Pig-to-NHP xenograft rejection follows three main temporal patterns: hyperacute (HAR), acute humoral (AHXR), and acute cellular (ACXR), with chronic rejection in longer-surviving grafts. HAR occurs within hours via endothelial injury, thrombosis, and anti-Gal–mediated complement deposition; AHXR arises days later with vascular inflammation and platelet-fibrin microthrombi; ACXR features mononuclear infiltrates; chronic rejection presents as intimal thickening and fibrosis. Combined histology, immunofluorescence, and functional data are essential for accurate diagnosis and immunomodulatory guidance in xenotransplantation (103–105).

In renal xenografts, humoral and complement-mediated mechanisms predominate, producing thrombotic microangiopathy and capillary damage, while chronic rejection manifests as vascular thickening and interstitial fibrosis (106). Current Banff criteria may need refinement to encompass complement-driven and innate immune–mediated lesions specific to xenotransplantation.

Thrombotic microangiopathy (TMA) is a frequent and clinically significant feature of xenograft injury, reflecting persistent coagulation incompatibilities between species and contributing to graft dysfunction and loss.

## 5 Molecular overlaps and differences between humans and swine

### 5.1 Comparison of human and porcine HLA systems

These clinical and pathological observations are grounded in molecular features, particularly those related to rejection and immunosuppression. HLAs and SLAs constitute the MHC in humans and pigs, respectively. They play central roles in antigen presentation and immune recognition. Advances in genetic engineering, including the generation of triple-KO pigs deficient in major carbohydrate antigens, have highlighted SLA molecules as residual xenoantigens and potential barriers to successful xenotransplantation (107). Both systems share a fundamental structural organization, comprising highly polymorphic class I and class II molecules essential for T cell activation (108, 109). Some epitopes present on SLA molecules are structurally similar to human HLA epitopes, which can lead to cross-reactivity of anti-HLA antibodies with porcine cells (110). Despite these molecular homologies, pigs also express unique carbohydrate xenoantigens (e.g., αGal, Neu5Gc, and Sda) absent in humans (111, 112). Another key divergence is the lack of expression of SLA class II-DP in pigs (107). Both systems are highly polymorphic, allowing broad peptide presentation but also contributing to strong immune responses against foreign tissues. Certain porcine SLA alleles, such as SLA-DRB106, can trigger particularly strong human T-cell responses, partly due to shared epitopes and cross-reactivity with human HLA alleles like HLA-DRB101 (113). Further commenting on both systems, Forneris et al. highlight their crucial role for antigen presentation and include similar components, such as β2-microglobulin, enabling cross-species antibody reactivity due to conserved domains. However, SLA-I functions as a xenoantigen in pigs, triggering T cell responses in human recipients, which poses a major barrier. Additionally, while human HLA-E and porcine SLA-I display about 70% sequence homology and comparable molecular sizes, certain human antibodies fail to cross-react with porcine counterparts, underscoring molecular divergences (114). Understanding the complex interplay of shared and unique antigenic determinants between HLA and SLA systems is critical for optimizing donor-recipient matching and developing strategies to mitigate immunologic rejection.

### 5.2 Differences between humans and pigs in HLA-G and its receptors

Recruitment of human NK cells to porcine tissues was first demonstrated in ex vivo pig organ perfusion studies in the 1990s (115). Subsequent *in vitro* work has clarified the molecular mechanisms underlying human NK cell adhesion and cytotoxicity against porcine endothelial cells. Strategies to overcome human anti-pig NK responses include blocking NK cell recruitment, expressing human MHC class I molecules on porcine cells to inhibit NK activation, and eliminating activating porcine ligands (94). To reduce human NK cell–mediated cytotoxicity, Cross-Najafi et al. engineered porcine endothelial cells to express human non-classical HLA class I molecules HLA-E and HLA-G which display immunoinhibitory activity while disrupting five genes encoding major xenoantigens and swine MHC class I molecules. Co-expression of HLA-E and HLA-G, confirmed by flow cytometry, significantly decreased human NK cell activation *in vitro* compared with cells expressing either molecule alone or parental cells, as assessed by CD107a expression. These modifications did not induce reactivity with human serum antibodies (116). CRISPR/Cas9 was used to generate GGTA1–/HLA-G1+ pigs by inserting HLA-G1 into the ROSA26 locus while deleting the GGTA1 gene. These pigs showed stable expression of the full-length HLA-G1 membrane-bound protein confirmed by sequencing and multiple assays in fibroblasts, organ tissues, and islets. GTKO/HLA*-G1+* fibroblasts reduced IFN-γ production and proliferation of T, B, and NK cells, and increased SHP-2 phosphorylation, suggesting effective immune modulation. Islets from these pigs reversed diabetes in mice without impairing function. HLA-G1 expression may help protect porcine xenografts from immune rejection (117). In humans, HLA-G receptors include the inhibitory immunoglobulin-like transcripts ILT2 (LILRB1) and ILT4 (LILRB2), as well as the killer cell immunoglobulin-like receptor KIR2DL4, which mediate its immunomodulatory functions on NK cells, T cells, and antigen-presenting cells, with KIR2DL4 being primarily expressed on NK cells (118). In pigs, leukocyte receptor complex contains a single KIR (KIR2DL1), an expanded set of inhibitory and activating LILRs, and a conserved novel inhibitory Ig-like receptor family (119).

### 5.3 Antibody-based and other available detection tools

Antibodies represent essential immunological tools that can be employed for the detection of porcine xenograft rejection. Zhang et al. monitored anti-pig non-Gal IgG and IgM antibodies in serial plasma samples from the recipient of the first 10-gene-edited pig cardiac xenotransplant. Following induction immunosuppression, serum anti-pig antibodies sharply declined and remained low until postoperative day 47. Thereafter, both IgG and IgM levels rose markedly, paralleling increased troponin and culminating in xenograft failure. Hence, quantitative tracking of anti-pig antibody titers may provide a real-time, sensitive marker of impending antibody-mediated rejection (120). However, antibodies are not the only tools available. In another study, circulating pig-specific DNA (cpsDNA) was developed and validated as a novel, non-invasive biomarker to detect xenograft rejection and monitor response to immunosuppression. cpsDNA quantification was established in mouse and NHP models, shown to reflect complement-dependent cytotoxicity *in vitro*, and to correlate with xenograft loss *in vivo*. cpsDNA levels decreased significantly following rapamycin treatment, and dynamic cpsDNA tracking was feasible in pig-to-monkey artery patch transplantation. cpsDNA measurement may offer a sensitive, specific, and minimally invasive tool to guide early intervention (121).

## 6 Discussion

This review shows that converging advances in multigene-edited donor pigs, targeted immunosuppression, refined surgical techniques, and improved rejection monitoring have moved porcine xenotransplantation from experimental models to early clinical translation, with cautious optimism warranted (122). Porcine kidney xenotransplantation may be regarded as the most advanced model. Recent reports indicate that kidney xenograft survival in humans has now approached nine months under optimized immunosuppressive regimens (eGenesis 69-genomic-edit donor pigs) (123). In 2025, the FDA authorized the UKidney first-in-human clinical trial, led by United Therapeutics in collaboration with major U.S. transplant centers, to evaluate the safety and function of genetically modified porcine kidneys transplanted into patients with end-stage renal disease (124).

This stage of progress raises ethical questions, such as the selection of candidates for these trials (125). Despite encouraging early results, major mechanistic obstacles persist, including incomplete innate immune control, coagulation incompatibilities, and uncertainties in long-term physiological integration. A profound ethical tension might arise between the urgent need to save lives amid organ shortages and the duty to protect highly vulnerable patients from disproportionate medical risks and experimental uncertainty (126). These issues are particularly exacerbated in pediatric patients due to their developmental immunologic immaturity, high vulnerability to severe and unpredictable infections under intense immunosuppression, profound challenges to valid informed consent under parental distress, uncertain long-term graft growth and sensitization risks (127). Regarding the first-in-human cardiac xenotransplantation trials, a four-criterion ethical framework to guide patient selection was proposed. It relies on medical need, capacity to benefit, patient choice, and compliance (128).

Major mechanistic obstacles persist. Incomplete innate immune control, particularly macrophage-mediated inflammation and NK cell cytotoxicity, remains inadequately addressed by current genetic modification strategies. Coagulation incompatibilities between porcine and human thromboregulatory systems continue to drive TMA in a substantial proportion of recipients, despite the integration of THBD and PROCR transgenes and anti-C5 complement inhibition. Uncertainties regarding long-term physiological integration, including porcine erythropoietin–human erythropoiesis mismatches, renin–angiotensin–aldosterone system discordances, and organ growth regulation in GHR-KO donors, have not been resolved in available follow-up periods. Furthermore, the absence of a validated Banff-equivalent classification system for xenograft histopathology, and the lack of standardized long-term surveillance protocols for cpsDNA and anti-pig antibody titers, represent important infrastructure gaps that must be addressed as the field advances toward larger clinical cohorts. To contextualize current progress and future prospects, **Table 2** summarizes the major barriers already overcome, the challenges currently being addressed in clinical trials, and the key scientific, ethical, and regulatory milestones still required for the successful clinical translation of porcine xenotransplantation.

**Table 2:** Current status, unresolved barriers, and translational priorities in porcine xenotransplantation.

Barrier/challenge	Status & key evidence	Remaining gap	Target horizon
Barriers overcome (2020–2024)			
Hyperacute rejection	Eliminated by triple xenoantigen KO (GGTA1, CMAH, B4GALNT2) and insertion of human complement regulators (CD46, CD55, CD59). First demonstrated in NHP models, subsequently confirmed in human decedent cases	Residual anti-non-Gal antibodies in sensitized recipients; need for pre-transplant antibody depletion in selected patients	(largely) Achieved
Acute vascular rejection	Markedly reduced by costimulation blockade (anti-CD40/CD154) combined with multigene-edited donors. Consistent NHP survival >200 days reported	Incomplete control of residual adaptive immune pathways; calcineurin inhibitor toxicity when combined with co-stimulation blockers	(largely) Achieved
Surgical feasibility	Validated for heart, kidney, and liver xenotransplantation in decedent and living human models. Heterotopic and orthotopic approaches described	Anatomical variation in donor pigs (GHR KO) may complicate vascular anastomosis; size matching optimization ongoing	(largely) Achieved
Partially controlled, active clinical trials			
Late AMR	Anti-pig IgG/IgM rebound observed at day 47+ in first cardiac recipient Rituximab-based B-cell depletion and complement inhibitors partially effective	No validated protocol to prevent late humoral rebound. Circulating pig-specific DNA (cpsDNA) emerging as early biomarker	Short to medium term
Coagulopathy/TMA	Coagulation incompatibilities reduced by THBD/PROCR transgenes and anticoagulation. TMA remains frequent	Species-specific differences in thromboregulatory proteins not fully corrected by current transgenic strategies. Anti-C5 complement inhibition now standard.	Short to medium term
Long-term renal graft survival (>6 months)	~9 months achieved in UKidney trial with 69-gene-edited donor (eGenesis). FDA authorized first-in-human trial 2025	Phase I trial still recruiting; long-term outcomes beyond 1 year unknown; sensitization risk in failed xenograft recipients not quantified.	Ongoing
Rejection monitoring	Multi-omics profiling defined early molecular predictors: CXCL9/10/11 signaling, plasmablast expansion, complement C3a in aqueous humor	No validated Banff-equivalent classification for xenograft histology. Serial cpsDNA and anti-pig antibody titer monitoring not yet standardized.	Short to medium term
Major unresolved barriers, horizon > 2030 +?			
Innate immune control	Macrophage-mediated inflammation and NK cell cytotoxicity persist despite genetic modifications. HLA-E/HLA-G expression on porcine cells reduces NK activation *in vitro*	*In vivo* suppression of innate pathways without global immunosuppression remains unsolved. PD-L1 transgenic pigs under investigation.	Medium to long
Durable immune tolerance	Mixed hematopoietic chimerism and thymic xenotransplantation show promise in NHP. Pig-to-pig allotransplantation validates tolerance induction strategies.	No protocol yet reliably induces donor-specific tolerance in pig-to-human setting without chronic immunosuppression.	Medium to long
Physiological incompatibilities	Porcine EPO inadequately stimulates human erythropoiesis; RAAS and ADH mismatches documented in NHP kidney xenotransplants	Organ-specific physiological gaps (hepatic coagulation factor production, renal electrolyte handling) not corrected by current genetic strategies.	Medium to long
Infectious safety (PERVs/zoonoses)	PERV inactivation achieved by CRISPR in 69-gene-edited pigs (eGenesis). Designated pathogen-free facilities required	Long-term surveillance for cross-species transmission lacking; regulatory frameworks for PERV-inactivated xenografts still evolving.	Medium to long
Systemic challenges: ethics, regulation & governance			
Patient selection criteria	Four-criterion ethical framework proposed for cardiac xenotransplantation: medical need, capacity to benefit, patient choice, compliance	Pediatric patients pose unique challenges: developmental immune immaturity, informed consent under parental distress, long-term sensitization risk	Ongoing
Long-term surveillance frameworks	Multi-omic profiling and cpsDNA tracking established as research tools	No international consensus on mandatory post-transplant monitoring endpoints. Banff xenotransplantation classification refinement underway.	Short to medium term
Regulatory harmonization	FDA authorized UKidney trial (2025); eIND pathway used for cardiac cases. IXA position paper on kidney xenotransplantation published	EMA framework for xenotransplantation lags behind FDA. International harmonization of GMP standards for DPF pig facilities required.	Short to medium term
Human-pig chimerism ethics	Transient human cell chimerism demonstrated in pig models. Public acceptance studies indicate readiness for oversight-guided research	Degree of humanization raising ethical concerns on animal moral status. International governance frameworks still under development	Short to medium term

Beyond the scientific barriers discussed above, recent U.S. experience also suggests emerging structural trends that may shape future clinical deployment. As CRISPR-Cas9 has substantially reduced the cost and complexity of multigene editing, major transplant centers or academic consortia may progressively establish dedicated in-house breeding programs for genetically engineered donor pigs, reducing dependence on a limited number of commercial suppliers. In parallel, the pig is likely to remain the preferred donor species not only for physiological compatibility, but also from an ethical and societal standpoint, particularly when compared with NHP.

The main limitations of this study are its narrative design, without a formal critical appraisal of the methodological quality of the studies included, and the fact that much of the available evidence relies on heterogeneous preclinical models. Conclusions are limited due to differences in the genes that were knocked in or knocked out. However, this approach is inherent to the objective of narrative reviews, which aim to provide a broad and integrative overview of a research field rather than to perform quantitative analyses.

Priority areas for the next phase of clinical translation include (1): achieving durable innate immune control through targeted genetic and pharmacological strategies (2); resolving coagulopathy via improved thromboregulatory transgene combinations (3); establishing validated Banff-equivalent histopathological criteria for xenograft rejection (4); standardizing cpsDNA and anti-pig antibody surveillance protocols; and (5) advancing regulatory harmonization between FDA and EMA frameworks to support multinational clinical trials.

## 7 Conclusion

Porcine xenotransplantation is transitioning from experimental innovation to clinical feasibility. As early human trials demonstrate encouraging short-term graft function, the pig may increasingly serve as both a donor species and a recipient model, potentially bridging fundamental immunological research with translational application. The next frontier may lie in achieving durable tolerance, refining management of innate immune and coagulation barriers, and establishing rigorous long-term surveillance frameworks to ensure sustainable clinical translation. International collaboration, regulatory harmonization, and the integration of ethical governance into clinical program design will be indispensable to realizing the full potential of xenotransplantation as a transformative response to the global organ shortage.
